# Resilience, and Personality Traits as Independent Correlates of Perceived Treatment Management Abilities in Middle-Aged and Older People Living with HIV

**DOI:** 10.1007/s10461-025-05010-4

**Published:** 2025-12-25

**Authors:** Shakaye R. Haase, David E. Vance, Andrea Wells, Cierra Hopkins Smith, Bulent Turan, Olivio J. Clay, Michael Crowe, Pariya L. Fazeli

**Affiliations:** 1 Department of Psychology, University of Alabama at Birmingham, Birmingham, AL USA; 2 School of Nursing, University of Alabama at Birmingham, Birmingham, AL USA; 3 University of Alabama at Birmingham Hospital, Birmingham, AL USA; 4 Center for Neurodevelopmental and Imaging Research, Kennedy Krieger Institute, Baltimore, MD USA; 5 Department of Psychology, Koc University, Istanbul, Turkey

**Keywords:** Resilience, Personality, Locus of control, Treatment management ability, HIV, Resiliencia, Personalidad, Locus de control, Capacidad de gestión del tratamiento, VIH

## Abstract

As people living with HIV (PLWH) age, they face various stressors that may negatively affect HIV treatment adherence and management. Resilience and personality characteristics have demonstrated associations with better health outcomes among people with various health conditions. The present study examines the association between dispositional resilience, the Big Five personality traits (openness, conscientiousness, extraversion, agreeableness, neuroticism), and locus of control with self-reported perceived HIV treatment management ability among middle-aged and older PLWH. This cross-sectional study included 174 PLWH aged 40 years and older. Treatment management ability was measured by a composite of three HIV health-related perceived treatment management ability measures. After conducting correlations to determine independent variables, a multivariable linear regression predicting treatment management ability was conducted with resilience, all Big Five personality traits, locus of control, and conceptually relevant covariates (health literacy, race, SES, substance use, depression), using a stepwise approach to determine the optimal number of predictors. The overall model predicting treatment management ability was significant, (*F*(5,142) = 20.348, *p* < .001, Adjusted *R*^2^ = 0.40), and this model retained resilience, openness, depression, and health literacy. Follow up mediation analysis found that resilience partially mediated the association between openness and treatment management ability. Resilience, openness, depression, and health literacy are important psychosocial factors related to treatment management ability. Interventions targeting resilience, openness to new information, depression, and health literacy may improve HIV treatment management abilities and ultimately health outcomes among older PLWH.

## Resumen

A medida que las personas con VIH envejecen, se enfrentan a diversos factores estresantes que pueden afectar negativamente la adherencia y el manejo del tratamiento. La resiliencia y las características de personalidad han demostrado estar asociadas con mejores resultados de salud en personas con diversas afecciones. El presente estudio examina la asociación entre la resiliencia disposicional, los cinco grandes rasgos de personalidad (apertura, responsabilidad, extroversión, amabilidad, neuroticismo) y el locus de control con la capacidad percibida de manejo del tratamiento del VIH, según autoinforme, entre personas con VIH de mediana edad y mayores. Este estudio transversal incluyó a 174 personas con VIH de 40 años o más. La capacidad de manejo del tratamiento se midió mediante una combinación de tres medidas de capacidad percibida de manejo del tratamiento relacionadas con la salud del VIH. Tras realizar correlaciones para determinar las variables independientes, se realizó una regresión lineal multivariable que predecía la capacidad de manejo del tratamiento con la resiliencia, los cinco grandes rasgos de personalidad, el locus de control y covariables conceptualmente relevantes (alfabetización en salud, raza, nivel socioeconómico, consumo de sustancias, depresión), utilizando un enfoque gradual para determinar el número óptimo de predictores. El modelo general que predijo la capacidad de gestión del tratamiento fue significativo (F(5,142) = 20,348, *p* < .001, R² ajustado = 0.40), y este modelo mantuvo la resiliencia, la apertura, la depresión y la alfabetización en salud. El análisis de mediación de seguimiento reveló que la resiliencia mediaba parcialmente la asociación entre la apertura y la capacidad de gestión del tratamiento. La resiliencia, la apertura, la depresión y la alfabetización en salud son factores psicosociales importantes relacionados con la capacidad de gestión del tratamiento. Las intervenciones dirigidas a la resiliencia, la apertura a nueva información, la depresión y la alfabetización en salud pueden mejorar la capacidad de gestión del tratamiento del VIH y, en última instancia, los resultados de salud en las personas mayores con VIH.

Modern antiretroviral therapies (ART) and improved healthcare have expanded treatment options for people living with HIV (PLWH) [1, 2]. PLWH are living longer, have access to better HIV healthcare, have a greater chance of becoming virally suppressed, experience less medication side effects, and have a stronger immune system than in prior decades [1, 3, 4]. Current estimates suggest that over 73% of the U.S. HIV population will be 50 years and older by 2030 [5]. HIV is now considered a chronic disease; therefore, care is focused on helping individuals manage the disease throughout their lifespan [2].

Assessing the construct of perceived HIV treatment management is an important outcome among PLWH as research supports such measures are sensitive to real-world treatment outcomes such as viral suppression [6–8], higher CD4 count, and self-reported medication adherence [7, 8]. The HIV Treatment Adherence Self-Efficacy Scale (HIV-ASES) [8], Beliefs Related to Medications (BERMA) scale [9], and Perceived HIV Self-Management Scale (PHIVSMS) [10] are three measures that have been consistently used across the literature to operationalize the construct of HIV treatment management abilities. These measures broadly assess an individual’s perceived self-efficacy and ability to adhere to and implement different aspects of their treatment such as medication adherence, symptom management, and engagement with health care providers.

PLWH are more likely than people without HIV to experience psychosocial risk factors such as mental health issues, substance use, poverty, racial inequality, HIV-related stigma, discrimination (e.g., race, LGBTQI+, class), unstable community infrastructure, housing issues, limited access to health care [11, 12], and trauma [13]. These factors are consistent sources of stress for PLWH and, in turn, may contribute to negative health outcomes such as medication non-adherence, failure to achieve viral suppression, low CD4 count, and low quality of life (QoL) [11, 12]. Research among middle-aged and older PLWH also highlights unique age-related challenges such as comorbid chronic illnesses, neurocognitive difficulties, limited social support, loneliness, and poor mental health [3, 14, 15]. Age-related illnesses that are comorbid with HIV (e.g., hypertension, diabetes, renal failure, cardiovascular disease, frailty) often present earlier and are more prevalent, compared to the general population [14, 16–18]. These unique factors experienced by middle-aged and older PLWH could contribute to overall stress and daily hassles which may negatively affect HIV health outcomes [19].

Yet, many middle-aged and older PLWH adapt to these various stressors, successfully age with HIV, and have positive HIV health outcomes [20–22]. Resilience resources may attenuate the effects of stress and strain on health outcomes. Among these resources are dispositional resilience [23, 24], personality characteristics [25], and locus of control [26], which have all been linked to positive health outcomes among various disease populations. Resilience has often been defined as the ability to bounce back from adversity [27]. Resilience has been positively associated with various HIV health outcomes such as adherence and lower viral load among PLWH [28], as well as QoL [29, 30], and cognitive function [31].

Personality traits may also influence adaptation to stressors and diseases. The Big Five personality traits (openness, conscientiousness, extraversion, agreeableness, and neuroticism) are one of the most consistently studied models of personality; however, the study of personality traits and HIV health outcomes (e.g., adherence, viral load) is limited [32, 33]. Conscientiousness has been one of the most consistent traits showing positive associations with health outcomes among individuals with various health conditions (e.g., diabetes, HIV, coronary heart disease), with mixed findings for neuroticism and less support for openness, extraversion, and agreeableness [33]. Goodwin and Friedman [34] found that in a nationally representative sample, conscientiousness was associated with the decreased likelihood of physical and mental health disorders, neuroticism was associated with increased likelihood, while less consistency was observed for agreeableness, extraversion, and openness. O’Cleirigh et al. [35] found that conscientiousness was the only Big Five trait associated with engagement in HIV case management services. In another study, higher conscientiousness was associated with lower viral load, increased CD4 count, and medication adherence over one-year among PLWH [32]. In a four-year longitudinal study, Ironson et al. [33] found that higher openness, extraversion, and conscientiousness were all associated with slower disease progression among PLWH. In a six-year follow-up study of older PLWH, only higher neuroticism was associated with HIV medication nonadherence [36].

Locus of control is another important factor that may impact treatment management abilities. Locus of control is generally regarded as the feeling that one can control the events that influence one’s life and it is often conceptualized as a facilitator of positive health behaviors [26, 37]. Individuals with higher levels of locus control are generally more likely to engage in health promoting behaviors. Prior research on internal locus of control noted positive associations with general health and health behaviors [26, 38] in a national population-based sample. In their systematic review, Náfrádi et al. [37] found overall support for the positive association between internal locus of control and medication adherence among individuals with several different health conditions (e.g., HIV, diabetes, asthma). Despite general support for the association between locus of control and health behaviors, specific research on health outcomes of PLWH such as medication adherence and viral load is limited, with some studies showing positive associations [39] or no significant associations [40, 41] with HIV medication adherence.

To our knowledge only a few studies have attempted to comprehensively assess PLWH’s ability to manage their health outcomes [31, 42], and limited studies investigating the role of personality characteristics [33], and locus of control [40]. Although the association between resilience and positive health outcomes of PLWH has strong support in the literature, given the aging population of PLWH more research is needed among middle-aged and older PLWH [20]. The purpose of this study was to investigate the associations between psychosocial protective factors including resilience, personality, and locus of control on perceived treatment management abilities in middle-aged and older PLWH while accounting for established risk factors that are negatively associated with HIV health outcomes such as depression [43–45], low health literacy [42, 46–48], substance use [49–51], socioeconomic status [52, 53], and race [54, 55]. Understanding the psychosocial factors associated with HIV health outcomes can influence the design of interventions, health care policy, and practice.

## Methods

### Participants and Procedure

The present study included baseline data from 174 PLWH aged 40 and older, who were recruited from a university HIV clinic to participate in a larger R01 parent study [56]. Participants were recruited by trained research assistants and screened using instruments developed by the study’s principal investigators and electronic medical records provided by the clinic. Inclusion in the study was determined based on a brief telephone screen, and eligible participants must not have self-reported any major neurological conditions or mental impairments (e.g., schizophrenia, Alzheimer’s disease), no head injury resulting in a loss of consciousness greater than 30 min, and not have been currently undergoing chemotherapy or radiation. The telephone screen also asked participants if they can read and write in English. Eligible participants were invited to the research lab, and after providing informed consent, participants completed a comprehensive battery of paper and pencil measures administered by research assistants. Approval of the study was granted by the Institutional Review Board (protocol #s F160122002 & IRB160601006), at the University of Alabama at Birmingham.

### Measures

A socio-demographic questionnaire was used to collect basic information such as age, race, sex, education level, and annual household income. Participants provided urine samples which screened for illicit substances: amphetamines, THC, methamphetamine, opiates, and cocaine. Participants were not excluded if tested positive for these substances. For analysis we used a binary variable indicating if participants were positive or negative for any of these substances. To measure socioeconomic status (SES), a composite *z* score was created between years of education and annual household income due to the conceptual and statistical (Spearman’s rho = 0.47) overlap between these variables.

#### Resilience

The Connor Davidson Resilience Scale 10 item (CDRS-10) is a 10-item measure of dispositional resilience/hardiness and the ability of individuals to adapt when faced with stress and adversity [27, 57]. The items consist of Likert-type questions with responses ranging from 0 = *“not true at all”* to 4 = *“true nearly all the time.”* Example items are: “I am able to adapt when changes occur” and “I tend to bounce back after illness, injury or other hardships”. This scale provides an overall score ranging from 0 to 40, with higher scores indicating higher levels of resilience. The CDRS-10 had good reliability in the present study, with Cronbach’s alpha α = 0.89.

#### Personality

The Big Five Inventory (BFI) [58] measures personality characteristics along five broad domains in accordance with the five-factor model of personality traits: openness (e.g., is curious about many different things), conscientiousness (e.g., does a thorough job), agreeableness (e.g., is generally trusting), extraversion (e.g., is outgoing, sociable), and neuroticism (e.g., worries a lot). The scale consists of 44 short phrases with responses ranging from 1 = *“disagree strongly*”, 3 = *“neither agree nor disagree”*, and 5 = *“agree strongly.”* This scale provides an overall score for each personality trait, with higher scores indicating being higher in that trait. The Cronbach’s alpha across the subscales ranged from α = 0.58 to 0.77 in the present study (openness = 0.74, conscientiousness = 0.71, extraversion = 0.58, agreeableness = 0.73, neuroticism = 0.77).

#### Locus of Control

Locus of control was measured using the Personality in Intellectual Aging Contexts (PIC) Inventory Control Scales Short Form [59, 60]. The PIC short form is a six-item measure that assesses an individual’s beliefs and attributions about their cognitive and intellectual functioning associated with everyday situations. The PIC has three domains: Internal (responsibility or changes in intellectual functioning lies within one’s own control); Chance (the belief that changes in intellectual abilities are due to external forces) and Powerful Others (reliance on others to accomplish intellectual tasks due to beliefs that others are better at those tasks). Items include “It’s up to me to keep my mental faculties from deteriorating”, “I have little control over my mental state”, and “I wouldn’t be able to figure out postal rates on a package without the postman’s help.” Responses are measured on a Likert-type scale with responses ranging from 1 = *“strongly agree”* to 6 = *“strongly disagree.”* This scale provides a total score ranging from 6 to 36, with higher scores indicating higher internal locus of control of cognitive functioning. The Cronbach alpha was α = 0.70, indicating good reliability.

#### Health Literacy

Four validated measures were used to generate a comprehensive health literacy composite reflective of both reading and numeracy, which has been previously found to be associated with treatment management abilities among PLWH [31, 42]. A sample-based *z* score was calculated for each of the four literacy measures, which was used to create a health literacy *z* composite (average) with higher scores reflecting better health literacy. The Test of Functional Literacy in Adults (TOFHLA) Reading Comprehension subtest [61] is a 50-item measure to test individuals’ ability to read and understand health information, with higher scores reflecting better health literacy. The Cronbach alpha for the TOFHLA was α = 0.74, indicating strong internal reliability. The Rapid Estimate of Adult Literacy in Medicine (REALM) is a 66-item measure to assess the ability to recognize medical terms (e.g., flu, asthma, prescription, osteoporosis) [62], with higher scores reflecting better ability. Only the total scores for the REALM were calculated (individual items were not entered), therefore the Cronbach alpha could not be generated. The Newest Vital Sign (NVS) [63] is a brief assessment tool in which respondents answer six items based on an ice cream nutrition label (e.g., “If you eat the entire container, how many calories will you eat?”). Higher scores indicate greater health literacy. The Cronbach alpha for the NVS was α = 0.69, indicating strong internal reliability. The Expanded Numeracy Scale (ENS) [64] is a seven-item measure of numeric ability in the context of health risk (e.g., “Which of the following numbers represents the biggest risk of getting a disease? 1 in 100, 1 in 1000, 1 in 10”). +Higher scores indicate greater understanding of health risks. The Cronbach alpha for the ENS was α = 0.94, indicating strong internal reliability. The mean correlation between the health literacy measures was *r* =.55.

#### Depressive Symptoms

The Center for Epidemiologic Studies Depression Scale Revised (CESD-R) [65] is a self-report measure of depressive symptoms within the general population. The CESD-R consists of 20 items which examine nine different symptoms of depression as defined by the Diagnostic and Statistical Manual of Mental Disorders, fifth edition (DSM-V) (sadness, loss of interest, appetite, sleep, thinking/concentration, guilt [worthlessness], tired [fatigue], movement [agitation] and suicidal ideation). Respondents are asked how often they have experienced each symptom in the past two weeks. Examples of questions on the CESD-R are: In the past two weeks I have felt “I felt depressed” and “Nothing made me happy.” Responses were scored on a 5-point Likert-type scale, with answers ranging from 0 = *“not at all or less than one day”* to 4 = *“nearly every day for 2 weeks.”* Scores on the CESD-R range from 0 to 60 and higher scores suggest more symptoms of depression. The Cronbach alpha for the CESD-R was α = 0.77, indicating strong reliability in the present study.

#### Perceived Treatment Management Abilities

Participants completed three different HIV health-related treatment management ability measures to generate a comprehensive composite. The HIV Treatment Adherence Self-Efficacy Scale (HIV-ASES) [8] is a 12-item scale that measures patient confidence to adhere to important treatment-related behaviors (e.g., medication adherence, following nutrition plans) when faced with potential barriers. Responses are measured on a Likert-type scale with responses ranging from 0 = *“cannot do at all”*, 5 = *“moderately certain can do”* and 10 = *“completely certain can do.”* This scale provides an overall score of adherence self-efficacy with scores ranging from 0 to 120. Higher scores indicate a greater confidence in adhering to one’s treatment plan. The Cronbach alpha of the HIV-ASES was α = 0.94, indicating strong reliability. The 53-item Beliefs Related to Medications (BERMA) scale [9] assesses self-efficacy across three domains: Memory for Medications (e.g., “I am good at remembering the amount of medication I need to take”), Dealing with Health Professionals (e.g., “I am good at dealing with health professionals for the purposes of my own health care”), and Attitudes About Medications (e.g., “My medications are the correct dosage for my medical conditions”). Responses are on a Likert scale ranging from 1 = *“strongly disagree”* to 5 = *“strongly agree”* such that higher scores reflect better abilities to manage and adhere to medication and healthcare, with a possible score range of 1 to 265. The Cronbach alpha of the BERMA total score for the present study is α = 0.96, indicating strong reliability. Perceived HIV Self-Management Scale (PHIVSMS) [10] is a 8 item measure of disease self-management self-efficacy. Items include “I am able to manage things related to my HIV infection as well as most other people” and “I handle myself well with respect to my HIV infection.” Responses are measured on a Likert-type scale with responses ranging from 1 = *“strongly disagree”* to 6 = *“strongly agree.”* Scores range from 1 to 40 with higher scores indicate a greater ability to manage different aspects of living with HIV. Cronbach alpha of the PHIVSMS for the present study is α = 0.90, indicating strong reliability. These measures conceptually overlap and have strong associations in prior research; therefore, a composite was created to reflect perceived treatment management abilities guided by prior work [31, 42]. A sample-based *z* score was calculated across the three HIV management measures, which was used to create a composite *z* score (average) with higher scores reflecting better treatment management abilities. The correlation between BERMA and PHIVSMS was ρ = 0.71, BERMA and HIV-ASES was ρ = 0.46, and PHIVSMS and HIV-ASES was ρ = 0.33.

### Statistical Analysis

Statistical analyses were performed using IBM SPSS Statistics for Windows (Version 30.0.0.0 Armonk, NY: IBM Corp). The sample included 174 PLWH from a larger parent study, therefore given that this was a convenience sample using secondary data from an existing study, no formal power calculation was conducted. Descriptive analysis was performed to summarize the data, followed by bivariate analysis (Pearson’s Correlation and T-test) between the main variables of interest. Following bivariate analysis, the Benjamini-Hochberg false discovery rate (FDR) procedure [66] was done to adjust the p-values and reduce Type 1 error rates for correlations between the independent variables of interest and potential covariate variables with perceived treatment management ability (a total of 14 bivariate correlations). Results from this procedure showed that all variables that were significant in the initial bivariate analysis also remained significant following the Benjamini-Hochberg adjustment. To determine significance, we used an FDR of 0.1 [67]. All the assumptions for regression analysis were tested, including collinearity and normality of residuals (errors). Results from the correlations were used to inform which variables would be included in the regression analysis. A multivariable linear regression predicting treatment management ability was conducted, including resilience, all Big Five personality traits, internal locus of control, and data-driven conceptually relevant covariates (health literacy, race, SES, substance use, depression), using a stepwise approach to determine the optimal number of predictors.

## Results

The study included 174 PLWH aged 40 years and older, with a mean age of 51.3 (*SD* = 6.9; range = 40 to 73). Most of the sample were male (62.1%) and identified as African American (85.6%). See Table 1 for sample characteristics.

**Table 1:** Descriptive characteristics among older people with HIV (*N* = 174)

Sociodemographic	
Age (years) *M (SD)*	51.3 (6.9)
Race	
White	14.4%
African American/Black or Other^a^	85.6%
Sex	
Male	62.0%
Female	38.0%
Education (years) *M* (*SD*)	12.6 (2.2)
SES composite *Z*-score *M* (*SD*)	− 0.67 (2)
HIV clinical variables	
Estimated duration of infection [EDI] (years)	16.7 (8.5)
Current CD4 (median[IQR])^b^	644 (379–858)
Nadir CD4 (median[IQR]) ^c^	254 (23–416)
Plasma viral load (% undetectable) ^d^	66.8%
Positive urine drug screen^e^	28.2%
Other variables	M (SD) Min-Max
Resilience	35.4 (9.3) 7–50
Openness	35.9 (6) 21–50
Conscientiousness	34.9 (5.6) 18–45
Extraversion	26.2 (4.9) 13–40
Agreeableness	36.6 (5.6) 19–45
Neuroticism	22.6 (6.5) 8–40
Locus of control	28.4 (5.9) 8–36
Depressive symptoms	17.4 (10.9) 0–49
Health literacy composite Z-score	− 0.01 (0.81)
Perceived treatment management Z-score	0.27 (0.39)

Data reported as mean (SD) for continuous variables unless stated otherwise; ^a^All African American/Black except 1 person reported ‘other’; ^b^*n*=124; ^c^*n*=157; ^d^*n*=146; ^e^positive test of at least 1 substance

### Main Analysis

Bivariate analysis revealed age, sex, and race were not associated with treatment management abilities. However, since race was associated at the trend level (t(170) = 1.769, *p* =.079; remained a trend after FDR correction), we retained it as a covariate in multivariable models. Higher SES (*r* =.38) and health literacy (*r* =.47) were associated with better treatment management abilities, while higher depressive symptoms were associated with poorer treatment management abilities (*r* = −.37). There was a significant difference between substance users and non-users on treatment management abilities (*t*(166) = 2.386, *p* =.018) such that substance users had poorer treatment management abilities. Resilience was associated with all Big Five personality traits (*r* ranges from − 0.16 to 0.34), internal locus of control (*r* =.27), and treatment management abilities (*r* =.45). Internal locus of control also had a positive association with treatment management abilities (*r* =.41), and all Big Five personality traits except for extraversion (*r* ranges from − 0.25 to 0.46). All Big Five personality traits were associated with treatment management abilities (*r* ranges from − 0.26 to 0.40). Associations between all relevant study variables are displayed in Table 2.

**Table 2:** Pearsons’s correlation matrix of study variables

	1	2	3	4	5	6	7	8	9	10	11	12
1. Age	1											
2. SES	0.18*	1										
3. Health literacy	− 0.06*	0.67**	1									
4. Depressive symptoms	− 0.07	− 0.22**	− 0.11	1								
5. Resilience	0.07	0.27**	0.28**	− 0.34**	1							
6. Locus of control	0.15	0.41**	0.44**	− 0.31**	0.27**	1						
7. Openness	0.17	0.44**	0.48**	− 0.12	0.31**	0.35**	1					
8. Conscientiousness	0.10	0.40**	0.38**	− 0.30**	0.34**	0.46**	0.38**	1				
9. Extraversion	0.03	0.12	0.13	− 0.27**	0.16*	0.10	0.21**	0.29**	1			
10. Agreeableness	0.06	0.17*	0.33**	−17*	0.20*	0.27**	0.27**	0.50**	0.31**	1		
11. Neuroticism	− 0.09	− 0.10	− 0.01	0.60**	− 0.37**	−25**	− 0.15	− 0.34**	− 0.24**	− 0.25**	1	
12. Treatment management	− 0.02	0.37**	0.47**	− 0.37**	0.45**	0.41**	0.40**	0.44**	0.16*	0.27**	− 0.26**	1

*p <.05; **p <.001

The stepwise multivariable linear regression predicting treatment management abilities included the following independent variables: resilience, all Big Five personality traits, internal locus of control, and conceptually relevant covariates (health literacy, race, SES, substance use, depression). The analysis revealed that the final model retained resilience, openness, depression, and health literacy as significant correlates of perceived treatment management ability (*F*(5,142) = 20.348, *p* <.001, Adjusted *R*^2^ = 0.40). Higher resilience (β = 0.22, *p* <.05), openness (β = 0.18, *p* <.05), and health literacy (β = 0.27, *p* <.001) had a positive association with treatment management ability, while depression (β = − 0.15, *p* <.05) had a negative association (see Table 3).

**Table 3:** Regression model showing significant associations with perceived treatment management abilities

	β^a^	SE	t	*p*
Resilience	0.22	0.003	2.99	< 0.005
Openness	0.18	0.005	2.29	< 0.05
Depression	− 0.15	0.003	−2.13	< 0.05
Health literacy	0.27	0.037	3.46	< 0.001

^a^Standardized beta

^*^Covariates in adjusted model were entered simultaneously and include race, sex SES, depression, substance use (i.e. positive test of at least 1 substance), and health literacy

### Posthoc Analysis

Given the results of the regression, we were interested in examining whether resilience and locus of control may explain the associations between personality (openness) and treatment management abilities. A parallel mediation model was tested to explore the role of resilience and locus of control in the association between openness and treatment management ability, adjusting for health literacy, race, sex, SES, substance use, and depression. In other words, do protective factors such as resilience and locus of control explain why individuals with higher dispositional openness have better treatment management abilities? The PROCESS macro program for SPSS was used for mediation analysis [68] and evidence for mediation is indicated if the confidence interval of the indirect effect does not include zero.

**Fig. 1:**
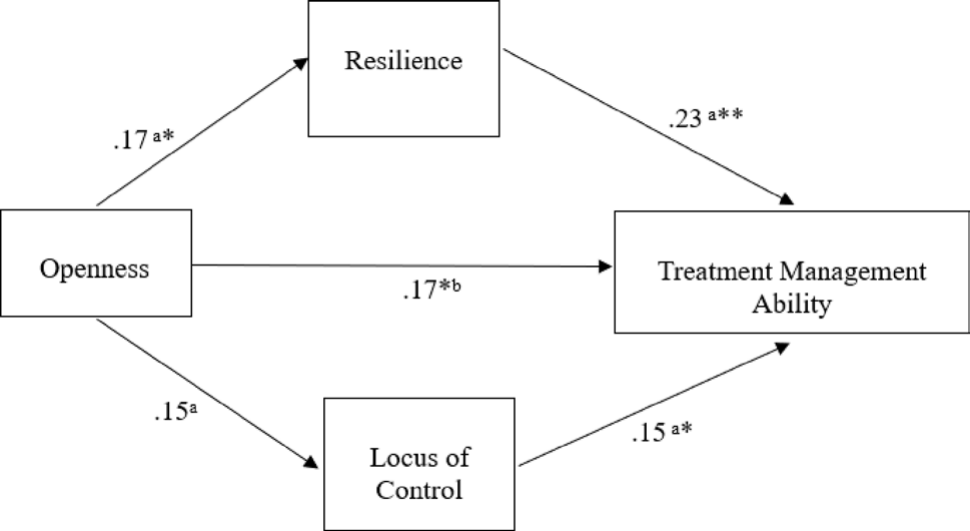
Regression coefficient for the association between openness and treatment management ability, mediated by resilience and locus of control. * *p* <.05; ** *p* <.01; health literacy, race, sex, SES, substance use, and depression were entered in the model as covariates; ^a^Standardized coefficient; ^b^Unstandardized coefficient

Mediation analysis revealed that openness had a significant positive association with resilience, resilience was positively associated with treatment management ability, and openness was positively associated with treatment management ability. Openness was not significantly associated with locus of control; however, locus of control had a significant positive association with treatment management ability (see Fig. 1).

The direct effect of openness on treatment management ability was significant (b = 0.012, SE = 0.005, 95% CI = 0.003 to 0.021). Importantly, the indirect effect of openness on treatment management ability through resilience was significant (β = 0.032, SE = 0.022, 95% CI = 0.001 to 0.088); however, the indirect effect was not significant though locus of control (β = 0.022, SE = 0.022, 95% CI = − 0.002 to 0.079). These findings suggest that resilience partially mediated the association between openness and treatment management ability, while locus of control had no mediating effect.

## Discussion

This present study examined the association between resilience, personality traits, locus of control, and perceived treatment management abilities among middle-aged and older PLWH, with a particular focus on intrapersonal protective factors that may have implications for intervention. Our final model, which considered statistically and conceptually relevant factors that may impact treatment management ability, showed that resilience, openness, depression, and health literacy were independent factors associated with treatment management abilities. Consistent with prior research [28], resilience continues to be an important correlate of health outcomes and overall well-being of middle-aged and older PLWH. Individuals with greater levels of resilience may be more likely to adjust to their HIV diagnosis, overcome age-related stressors (e.g. health comorbidities, frailty) and follow through with treatment regimen.

All Big Five personality traits were associated with perceived treatment management abilities at the bivariate level, with conscientiousness having the strongest association. This finding is consistent with prior research where conscientiousness has been the most consistent among the personality traits in predicting HIV health outcomes (lower viral load, increased CD4 count, and medication adherence) [35]. Yet, in multivariable analysis, openness was the only personality trait that had a significant association with treatment management ability, which suggests that the other personality traits may be less of a driver of treatment management abilities when accounting for other psychosocial factors such as resilience, locus of control, depression, and health literacy. Individuals who are more open to new experiences and information may be more likely to seek out information related to their HIV care and be more engaged in treatment [33].

Locus of control was significant at the bivariate level, but it was not retained in the overall model when accounting for other psychosocial variables. This finding suggests that the bivariate association was likely due to overlap between other predictors’ associations with perceived treatment management ability. Additionally, the present study examined locus of control for cognitive and intellectual functioning, which could be less sensitive to broad health outcomes compared to more general measures locus of control [26, 37].

While not being the direct focus of the present study, not surprisingly depression and health literacy emerged as significant predictors of treatment management ability both at the bivariate and multivariate level. The negative impact of depressive symptoms on HIV health outcomes has been consistent throughout the literature [45]. Symptoms such as persistent sadness, loss of energy/motivation, and difficulty concentrating can interfere with engagement and following through with HIV treatment. Heath literacy had the strongest positive association with treatment management ability compared to any other factors, which further supports previous research [48]. The ability to comprehend and utilize health information is very important in maintaining health. Middle-aged and older PLWH who have greater health literacy may find it easier to follow through with health regimens, manage any potential side effects, build a relationship with providers, and be more actively engaged in care.

Following initial analysis, the mediating role of resilience and locus of control in the association between openness and treatment management ability was investigated. Only resilience was found to be a partial mediator, suggesting that individuals who have higher openness traits, may be more resilient which may in turn support better self-efficacy for and adherence to treatment management. In other words, people with higher dispositional openness may have better HIV treatment management ability in part due to their higher levels of resilience.

### Implications for Practice

Results from this study have important implications for HIV health care practice. First, researchers could incorporate resilience building and teaching HIV health information in designing and testing interventions to improve HIV health management, which may be particularly needed in PLWH with depression and low health literacy. Health literacy had the strongest association with perceived treatment management abilities, which underscores the need for health care providers to spend adequate time on continuous HIV health education while delivering various services. Such interventions targeting resilience, depression and other psychosocial resources may impact treatment management abilities by improving self-efficacy and empowering individuals to better manage their disease and overcome barriers and stressors that can impact adherence behaviors. Resilience interventions incorporating cognitive behavioral coping skills and psychoeducation about managing stress have shown good feasibility and acceptability among PLWH [69, 70], but further work is needed to test their efficacy (and mechanisms) for improving treatment behaviors and outcomes.

### Strengths

To our knowledge this is one of the few studies that adopted a more comprehensive approach to studying HIV health behaviors among PLWH, conceptualized as perceived treatment management abilities. This composite measure of three established instruments (HIV-ASES, BERMA, PHIVSMS) incorporates the conceptual overlap among all three, and the unique elements assessed by each individual measure, and has been used in prior work [31]. The present study adds to the existing gaps in the literature, by highlighting resilience, openness, and health literacy as important psychosocial factors associated with treatment management ability of middle-aged and older PLWH. The present study also adds to the growing body of literature exploring the associations between personality characteristics and HIV health outcomes, which remains largely unexplored [33]. Our study was conducted in Alabama which has been classified by the Center for Disease Control and Prevention (CDC) as being one of the states in the “Deep South” which are disproportionately affected by HIV [12]. Investigating factors associated with better HIV disease management is particularly important within this region and to the national HIV response.

### Limitations

Participants were recruited from a local HIV clinic in Alabama and the findings from this study may not be generalizable to PLWH in other states or PLWH recruited from other settings. The range of the study sample (40–73 years old) consisted of participants in middle age and older adulthood, who are in a different developmental stage, and potentially experiencing different life concerns while living with HIV. The role of psychosocial factors and perceived treatment management abilities may differ, which may not be captured in the present study. The outcome of this study was self-reported perceived treatment management ability, which may not fully reflect actual treatment management behavior. The use of self-report measures may also be subject to social desirability, where participants may respond in a manner that presents them more favorably. In this study, no other objective data on treatment management behaviors such as doctor visits from medical records were collected.

The present study is cross sectional in design, which limits our ability to infer causal effects and does not account for other factors potentially associated with treatment management abilities. Research among middle-aged and older PLWH who are aging highlights factors such as frailty, medial comorbidity, loneliness, and lack of support as significant factors affecting their well-being. The present study utilizes stepwise regression analysis with a sample size of 174, which increases the risk of the final model “overfitting” based on the present data. Follow up research with a different and lager sample size and can corroborate the factors retained in our model (i.e. resilience, openness, depression, and health literacy).

### Future Research

Future research could use a longitudinal design to better predict treatment management abilities over time. Additionally, research should examine more complex associations such as mediating and moderating effects with other important variables such as stress, social support, and HIV stigma. Specifically, understanding the underlying behavioral and mental health mechanisms whereby resilience impacts treatment management abilities is an important area for future research. Prior research among PLWH highlight the prevalence of neurocognitive issues [71] and their association with HIV health outcomes such as adherence [72, 73] and viral load [74]. Research also supports the association between cognitive functioning and both resilience [75] and personality traits [76]. Future research can also explore neurocognitive factors as predictors of perceived treatment management abilities and examine the role of resilience and personality traits in these associations. Given the aging population of PLWH, more research is needed to specifically understand psychosocial factors and treatment management abilities of older adults aged 65 and older, as a specialized subgroup.

## Conclusion

The present study is one of few exiting research that adopted a comprehensive approach to studying HIV health behaviors among PLWH, conceptualized as perceived treatment management abilities. Our findings demonstrated that openness, personality trait, resilience, depression, and health literacy, are significant predictors of perceived treatment management ability. Follow up analysis demonstrated that resilience partially mediated the association between openness and perceived treatment management ability. This highlights the potential need to focus on these psychosocial factors when designing intervention to improve health outcomes for PLWH.

## Data Availability

The data supporting the findings of this study are available to qualified investigators upon request.
